# Commercial sexual exploitation exposure, food insecurity, and help-seeking attitudes in high school youth

**DOI:** 10.3389/fpsyt.2026.1901555

**Published:** 2026-10-21

**Authors:** Lisa M. Jones, Jennifer E. O’Brien, Vanessa Voller, Emily Boyd, Lauren Martin

**Affiliations:** 1 Crimes Against Children Research Center, University of New Hampshire, Durham, NH, United States; 2 School of Social Work, University of Texas Arlington, Arlington, TX, United States; 3 School of Nursing, University of Minnesota, Minneapolis, MN, United States

**Keywords:** exploitation, help seeking, structural vulnerabilities, trafficking, youth

## Abstract

**Introduction:**

Commercial sexual exploitation (CSE) of youth is a form of victimization that is shaped by socioeconomic conditions, yet more information is needed on how these conditions are related to differences in help-seeking attitudes for vulnerable youth.

**Methods:**

Self-report survey data were collected from a sample of 169 high school students from Houston, Texas. Cumulative risk analysis examined relationships between CSE exposure, financial insecurity, food insecurity, and help-seeking confidence and likelihood.

**Results:**

Financial instability was associated with greater likelihood of CSE exposure, with food insecurity emerging as a particularly strong correlate. Both CSE exposure and food insecurity were linked to lower confidence in seeking help and reduced likelihood of seeking assistance if confronted with a request to trade sex for money or something of value. These associations were driven primarily by a lower reported likelihood of seeking help from a parent or trusted adult.

**Discussion:**

Findings highlight the intersection of structural vulnerability and CSE risk among adolescents and suggest that youth experiencing both food insecurity and CSE exposure may face heightened barriers to help-seeking. Prevention and intervention efforts should ensure that youth are aware of and connected to trusted extra-familial sources of support within their schools and communities.

## Introduction

The commercial sexual exploitation (CSE) of children and youth is a significant public health and human rights issue globally and in the United States. CSE exposure is associated with a variety of mental health concerns including elevated rates of anxiety, depression, and post-traumatic stress disorder (PTSD) along with physical health consequences such as violence related injuries, unplanned pregnancy, sexually transmitted diseases (1). While CSE is sometimes used interchangeably with the term “sex trafficking,” trafficking terminology can sometimes imply third party involvement, physical victimization, or the harboring and transportation of victims. For the current paper, we use the broader term CSE to refer to incidents where an individual is induced to exchange sexual acts or images for something of value including money, food, shelter, drugs, transportation, or other goods or favors (2). In the U.S., force, fraud or coercion do not need to be established for cases involving individuals under age 18, as minors cannot legally consent to commercial sex under federal trafficking laws (3).

Prevalence rates for the CSE of children and youth are difficult to ascertain due to underreporting, stigma, and the hidden nature of exploitation, and rates vary substantially depending on the methodology, sample, and definition used in the study. Reviews find rates between 3-5%, and up to 10% among more vulnerable populations (e.g., unhoused youth, youth involved with child protective services) (4–6).

Although any young person is at risk for CSE, disparities in CSE are observed across intersecting axes of structural vulnerability at the individual, family, and community level (7). Structural determinants of health refer to the societal and political forces and hierarchies that influence how resources are distributed and affect health (8). These include economic inequality, racism and discrimination, housing instability, and barriers to health care and other service. Structural vulnerabilities can increase youth risk for CSE: higher rates of CSE are observed in BIPOC youth and youth who identify as LGBTQ2S+ (9, 10), youth with physical and cognitive disabilities (5), and youth experiencing homelessness (11). Structural vulnerabilities can also increase individual, familial, and community risks (e.g., drug use, community violence, interpersonal violence), placing already vulnerable youth at even greater risk for CSE (12).

A socio-ecological framework provides context to understand how structural vulnerabilities may contribute to youth exposure to CSE. This framework makes explicit that individual vulnerabilities and risks are imbedded within broader, interacting spheres of influence that include relationships (e.g., family, peers), communities, and society (e.g., structures, systems, policies, and cultural norms) (13). Economic inequality, racism, discrimination, and inequitable access to safety nets can constrain resources at the community and family levels. In turn, unmet material needs and limited access to resources at the community and family level may increase youth vulnerability to exploitation. Finigan-Carr et al. (14) describe how structural determinants of health affect sexual risk behavior. Consistent with this framework, research on CSE suggests that social and environmental factors may be more influential predictors of CSE than individual-level factors (15, 16).

### CSE and financial insecurity

Within the socio-ecological context, financial insecurity represents one pathway through which structural inequalities may contribute to youth vulnerability to CSE. Financial insecurity has substantial implications for health and well-being (17) and is closely intertwined with many of the adversities that place youth at risk for CSE (18, 19). Economic stress has been identified as a risk factor that is related to CSE risk for youth in rural, suburban, and urban communities (20). Economic stress may increase vulnerability to CSE through several pathways. Economic hardship may indirectly create circumstances associated with increased risk for CSE, through periods of homelessness for example (21). Traffickers and other exploitative individuals may also take advantage of unmet economic needs for youth or families by offering money, housing, food, transportation, or other needed resources. (22).

Less is known about the specific association of food insecurity and commercial sexual exploitation in the United States. Food insecurity affects nearly 13.7% of all households in the United States (23) (defined as difficulty providing food for all members of the household at some time during the year) and households with children are more likely to be food insecure than households without children (24). While food insecurity is high in lower income families, research identifies that food insecurity is also an issue in families with incomes above the poverty line (25). Food insecurity has been linked to significant negative health impact on children and adolescents (26). Research has also identified links between food insecurity and symptoms of depression and anxiety, and externalizing behaviors for youth (27, 28) and parental interpersonal violence (29, 30).

However, there has been little research specific to studying the role food insecurity plays in CSE risk in the United States. A keyword search of two of the leading journals about human trafficking (*Journal of Human Trafficking* and *Anti-Trafficking Review*) revealed no research on the intersections of food insecurity and CSE risk in the United States. One study of student data from Minnesota youth identified that boys who reported trading sex also reported higher rates of food insecurity in the last month among other risk factors (31). There has been research conducted in lower- and middle-income countries (LMIC) that finds traffickers target lower income households and individuals to manipulate acute food insecurity (32). In a study in Swaziland, hunger and acute food insecurity were cited as a as a primary motivation to engage in transactional sex for children (33). Another study in Ghana found that girls reported escaping acute and chronic food insecurity as one of the primary reasons for their involvement in commercial sex (34). A different study conducted with adult women engaged in transactional sex in Spain, Italy, and Portugal revealed that pandemic-induced food insecurity served as a primary driver for their entry into sex work (35). Building on this link between commercial sex and food insecurity, a recent commentary highlighted how the COVID-19 pandemic further exacerbated food insecurity among youth, thereby increasing their risk of CSE (36). Together, these findings underscore the urgent need for more research into the intersections of food insecurity and CSE.

### Improving youth help-seeking for CSE exposed youth

One of the key strategies for improving prevention and response to youth exposed to CSE is to increase the likelihood of help-seeking. Help-seeking actions by youth are result of a complex combination of knowledge, trust, past-experiences seeking help, and the availability of helping resources (37–39). Reaching out to trusted adults can serve a protective function by reducing CSE exposure, and for survivors it can help ensure access to support, safety, and mental and physical health care. A recent scoping review about help-seeking behaviors among youth who have experienced CSE found that universally youth are more likely to access informal help (e.g., family, peers) than formal services (40). This scoping review also found that many youth believed they only deserved help after experiencing physical violence and identified different help seeking patterns between older and younger youth. An analysis of data on a large sample of youth across the U.S. identified that older youth reported less intention to seek help than younger youth, and that gender minority youth and youth in juvenile justice settings reported lower rates of help-seeking intention as well compared to other youth (41). These findings suggest that the youth who may be most at risk for CSE are also the ones who report being less likely to seek help. Given the established link between financial insecurity and CSE risk, it is important to understand more about how the intersection of these conditions may be affecting help-seeking confidence and intentions for youth.

### Current study

There has been substantial progress toward improving the availability and quality of services for trafficking survivors, advancing a more victim-centered justice system, and expanding prevention efforts. However, meaningful reductions in CSE will ultimately require addressing the social and economic factors that place youth at risk. Research has begun to clarify the socioeconomic conditions associated with CSE, and the implications of these conditions for improving prevention and response. The current study contributes to this effort by examining the relationship between financial insecurity, food insecurity, CSE exposure, and youth attitudes toward help-seeking using survey data from a diverse group of high school students. Given our focus on help-seeking, we conceptualized CSE exposure broadly to capture youth proximity to and personal experience with CSE. This included knowing someone who is commercially trading sex, considering trading sex for money or other items of value, and direct involvement in CSE. We hypothesized that financial insecurity would be a predictor of CSE exposure, and that both CSE exposure and financial insecurity would negatively impact help-seeking self-efficacy and likelihood.

## Methods

### Sample

The data analyzed for the current study were collected from a convenience sample of 169 students at a high school in Houston, Texas, U.S. The data were collected as part of a larger study to gather psychometric data on a draft survey instrument for eventual use in an evaluation of a trafficking prevention program. The participating high school serves over 1,500 9^th^-12^th^ grade students. Fifty-two percent of students are Black and 46% are Latino, and approximately 90% of students are classified as being from socioeconomically disadvantaged backgrounds. **Table 1** provides data on sample demographics. The sample was roughly equivalent across high school grades with 25.3% in 9^th^ grade, 30.1% in 10^th^ grade, 21.7% in 11^th^ grade, and 22.9% in 12^th^ grade. The sample was also fairly evenly split between female (46.7%) and male students (53.2%). The majority of the sample described their sexual orientation as heterosexual (64.5%), with 35.5% of youth describing other sexual orientations (e.g., homosexual, bisexual, asexual, or other minority sexual orientations). Most students in the sample described their race/ethnicity as Black/African-American (61.9%) or Latino or Hispanic (33.9%), with less than 5% of the sample identifying with any of the following race/ethnic groups: Asian, Hawaiian/Pacific Islander, Native American, White, or “other” race/ethnicity.

**Table 1:** Bivariate subgroup differences in youth characteristics between CSE-exposed and non-CSE-exposed youth.

Youth characteristics	All youth (N = 169) % (n)	No CSE exposure (n=144) % (n)	CSE exposure (n=25) % (n)	X^2^	p
Age				>0.00	.98
14–16 years	56.2 (95)	56.2 (81)	56.0 (14)		
17–20 years	43.8 (74)	43.7 (63)	44.0 (11)		
Gender				0.32	.57
Male	46.7 (79)	45.8 (66)	52.0 (13)		
Female	53.2 (90)	54.2 (78)	48.0 (12)		
Race and ethnicity				1.36	.51
Black/African-American	61.9 (104)	61.5 (88)	64.0 (16)		
Hispanic/Latino	33.9 (57)	35.0 (50)	28.0 (7)		
Multiple or other race or ethnicity	4.2 (7)	3.5 (5)	8 (2)		
Sexual orientation				2.00	.16
Heterosexual	64.5 (109)	66.7 (96)	52.0 (13)		
LGBQ+	35.5 (60)	33.3 (48)	48.0 (12)		
Grade-level				0.97	.81
9^th^ grade	25.3 (42)	24.8 (35)	28.0 (7)		
10^th^ grade	30.1 (50)	29.1 (41)	36.0 (9)		
11^th^ grade	21.7 (36)	22.7 (32)	16.0 (4)		
12^th^ grade	22.9 (38)	23.4 (33)	20.0 (5)		
Food insecurity	33.8 (51)	27.9 (36)	68.2 (15)	13.63	**<.001**
Financial stress	44.4 (64)	42.5 (51)	54.2 (13)	1.10	.29

Bolded values identify p-values ≤.001; CSE, commercial sexual exploitation.

### Procedures

Surveys were administered in three health classes at the high school in January 2025. The school was provided with a $3,000 stipend for the help they provided with planning and data collection. Prior to survey administration, caregivers of students were sent a letter that provided detailed information on the study and the option to complete either a paper or online consent form. Signed caregiver consent was required for student participation. Prior to survey administration, on-site project staff read an assent form verbally to the youth. Survey data were collected during class via a secure online survey offered in both English and Spanish. Surveys took the students approximately 30 minutes to complete. Students who did not have parental consent to participate or did not assent to participation completed alternate computer work. All youth were provided with debriefing information following survey administration that included information about support options (e.g., school staff, local resources, national hotlines) if the survey questions brought up any concerns or discomfort. All procedures were approved by the Institutional Review Board (IRB) at the University of New Hampshire.

### Measures

The survey administered to youth participants included a range of scales being reviewed for the larger study, however, the current study focused on a set of scales and questions that asked respondents about and CSE exposure, financial insecurity, help-seeking confidence, and help-seeking likelihood. Information on these scales is included below:

Exposure to CSE. Youth were asked 5 yes/no questions about CSE exposure: 1) “Do you know or strongly suspect someone in your life is trading sex?” 2) “Have you considered trading sexual activity for money or other items of value?” 3) “Has someone asked you to trade sexual activity for money or other items of value?” 4) “Have you shared sexual photos, videos, or live streams online for money or other items of value?” 5) “Have you ever traded sexual activity for money or other items of value?” A composite, dummy-coded “CSE exposure” variable was created, with respondents coded as exposed if they endorsed any of the five items.

Financial insecurity. Respondents were asked 2 financial insecurity questions. A question about financial stress asked: “In the past 30 days, how often did you or your family not have enough money to pay one or more bills (e.g., electricity, rent, cable)?” A second question about food insecurity asked: “In the past 30 days, how often did you skip meals or eat less because you or your family didn’t have enough money for food?” Response options for both questions were: “never”, “rarely”, “sometimes”, “often”, “always” and “not sure.” Both financial instability questions were dichotomized with 0 = never, rarely, or sometimes and 1 = often or always.

Help-seeking confidence. Three questions asked participants about confidence that they would know where to go for help in three situations: 1) “If someone had nude or nearly nude pictures of you and was threatening to share them with friends and family members;” 2) “If you were feeling pressured to trade sexual activity for any reason including a place to stay, food, money, substances, rides, or other items of value;” and 3) “If you were at a party and felt unsafe.” Response options ranged from 1 to 5 (“not at all confident” to “very confident”). A composite “help-seeking confidence” score was calculated by averaging the three items.

Help-seeking likelihood. Youth were also asked questions about the likelihood of seeking help if they were “feeling pressured by somebody to exchange sex for money or other items of value.” Questions asked how likely they would be to: 1) handle it on their own; 2) get help from a parent or trusted adult; 3) get help from a friend, 4) look up advice on the internet, 5) get help from a crisis hotline or community agency, or 6) call 911/talk to police. Response options ranged from 1 to 5 (“very unlikely” to “very likely”). A composite “help-seeking likelihood” score was calculated by averaging the last 5 help-seeking items.

Youth demographics. Information was collected from youth on their age and grade-level (9-12^th^ grade). Racial/ethnic identity was collected across eight categories (Asian; Black/African-American; Hawaiian/Pacific Islander; Latino/Hispanic; Native American; White; and other race/ethnicity). Youth also reported on their gender (male, female, or other) and sexual orientation (heterosexual/straight, gay, lesbian, homosexual, bisexual, or other).

### Data analysis

Analyses were conducted using Stata 19.5 (42). Data were collected from a total of 196 youth participants, but cases with more than 80% missing data were dropped from analyses, with a resulting sample of 169 participating youth.

Descriptive and bivariate analyses were first used to compare youth with and without exposure to CSE on demographic characteristics and indicators of financial insecurity. Chi-square tests were used for categorical variables, with Fisher’s exact tests used when cell sizes were small. Next, bivariate linear regression models were used to examine whether CSE exposure was associated with differences in help-seeking confidence and likelihood of reporting to different sources. Finally, a four-level cumulative risk variable was created based on CSE exposure and food instability, representing increasing levels of vulnerability.

We examined whether cumulative risk level was associated with likelihood of disclosing CSE-related concerns to a parent or trusted adult. Linear regression models estimated the association between cumulative risk level and likelihood of disclosure to a parent or trusted adult, adjusting for age, gender, sexual orientation, and grade. Polynomial contrasts were used to test whether the adjusted means followed a linear pattern across cumulative risk levels.

## Results

Out of 168 respondents, 14.8%, reported exposure to commercial sexual exploitation (CSE) (n=25). This composite identified youth who said yes to any of the following: knows or strongly suspects someone in their life is trading sex (6%, n=10); considered trading sexual activity for money or other items of value (3%, n=5); was asked by someone to trade sexual activity for money or other items of value (9%, n=15); shared sexual photos, videos, or live streams online for money or other items of value (4%, n=6); and has ever traded sexual activity for money or other items of value (3%, n=4).

There were no demographic differences between groups: youth with and without CSE exposure in the sample did not significantly differ on age, gender, race and ethnicity, sexual orientation, or grade-level (see **Table 1**). There was also no difference between groups on a general measure of self-reported family financial stress. However, there were significant differences between groups on the measure of food insecurity. Youth with CSE exposure were substantially more likely to report food insecurity compared to non-CSE exposed youth (68.2% vs. 27.9%, χ^2^ = 13.63, p<.001).

### Differences in help-seeking confidence and likelihood by CSE exposure

**Table 2** presents group differences in help-seeking confidence and likelihood of seeking help for CSE risk from different helping sources. Compared to non-CSE exposed youth, youth exposed to CSE reported significantly lower confidence in seeking help in two of the three scenarios provided to participants. Compared to non-CSE-exposed youth, youth exposed to CSE reported significantly lower confidence in seeking help in scenarios where they felt unsafe at a party (B = -.491, p = .04, and if they were feeling pressured to trade sex (B = -.521, p = .03). Help-seeking confidence if someone was threatening to share sexual pictures was also lower among CSE-exposed youth, although the difference did not reach statistical significance (B = -.412, p=.11). A composite help-seeking confidence score based on all three scenarios was significantly different between CSE-exposed (M = 3.52, SD = 1.12) and non-CSE-exposed (M = 3.99, SD = 0.83) groups (B= -.472, p = .01).

**Table 2:** Differences in continuous outcomes by CSE exposure.

Outcome variable	All youth (N = 169) mean (SD)	No CSE exposure (n=144) % (n)	CSE exposure (n=25) % (n)	Coeff.	p
Help-seeking confidence					
Threats to share pics	3.63 (1.19)	3.69 (1.16)	3.28 (1.31)	-.412	.11
Pressured to trade sex	4.07 (1.34)	4.15 (1.05)	3.62 (1.34)	-.521	**.03**
Felt unsafe at a party	4.10 (1.10)	4.17 (1.04)	3.68 (1.34)	-.491	**.04**
All of the above	3.92 (0.89)	3.99 (0.83)	3.52 (1.12)	-.472	**.01**
Help-seeking likelihood for CSE risk					
Handle myself	3.26 (1.31)	3.22 (1.33)	3.48 (1.22)	.256	.37
Any help-seeking source	3.41 (0.87)	3.47 (0.84)	3.09 (1.07)	-.382	**.04**
Parent/trusted adult	3.79 (1.21)	3.94 (1.07)	2.96 (1.57)	-.977	**<.001**
Friend	3.54 (1.16)	3.56 (1.15)	3.40 (1.22)	-.160	.53
Internet	2.89 (1.27)	2.91 (1.27)	2.80 (1.30)	-.108	.70
911/police	3.27 (1.33)	3.32 (1.30)	2.96 (1.49)	-.364	.21
Community agency	3.56 (1.34)	3.61 (1.32)	3.25 (1.48)	-.363	.22

Bolded values identify p-values ≤.05; CSE, commercial sexual exploitation.

A second set of bivariate linear regression models analyzed respondents help-seeking likelihood for a scenario where someone was pressuring them to trade sex. There was not a statistically significant difference between groups in the likelihood that they would try to handle the situation themselves, However, overall there was a statistically significant differences between groups in the likelihood they would seek help from a helping source if being pressured to trade sex, with CSE-exposed youth reporting significantly less likelihood of seeking help (M = 3.09, SD = 1.07) than non-CSE-exposed youth (M = 3.47, SD = 0.84).

This difference appears to be predominantly driven by a significant difference between the likelihood of seeking help from a parent or trusted adult. CSE-exposed youth reported substantially lower likelihood of seeking help from a parent or trusted adult (M = 2.96, SD = 1.57) relative to non-exposed youth (M = 3.94, SD = 1.07, B = -0.977, p <.001). There were no statistically significant differences between groups on the likelihood of seeking help from friends, the internet, calling 911, or from a community agency. Most youth indicated they would seek help from parents, friends, or a community agency. Fewer indicated they would seek help from the Internet or 911/police.

### Cumulative risk model predicting disclosure to a parent or trusted adult

In order to understand more about how CSE exposure and food insecurity together affect the likelihood of seeking help from a parent or trusted adult, we used multivariate regression analysis to examine cumulative risk controlling for age, gender, sexual orientation, and grade level (see **Table 3**). Relative to youth with neither CSE exposure nor food insecurity, youth experiencing both CSE exposure and food insecurity reported significantly lower likelihood of disclosure to a parent or trusted adults (B = -.122, SE = 0.43, p = .005). Neither food insecurity nor CSE alone was significantly associated with help-seeking likelihood from parents/trusted adults. However, as seen in **Figure 1**, analyses identified a significant linear trend across cumulative risk groups (B = -0.44, SE = 0.15, p = .005), suggesting progressively lower likelihood of seeking help from a parent or trusted adults as structural vulnerability increases.

**Table 3:** Linear regression of likelihood of parent/trusted adult disclosure across cumulative risk groups.

Risk group	β	SE	t	p
*Reference group: No CSE + Food Secure*				
No CSE + Food Insecure	-0.07	.22	-0.34	.73
CSE + Food Secure	-0.37	.53	-0.69	.49
CSE + Food Insecure	-1.22	.43	-2.87	.005
Constant	4.03	.23	17.76	<.000

Models include controls for age, gender, sexual orientation, and grade. Robust standard errors reported. A significant linear trend across cumulative risk levels was observed (B = −0.44, SE = 0.15, p = .005).

**Figure 1:**
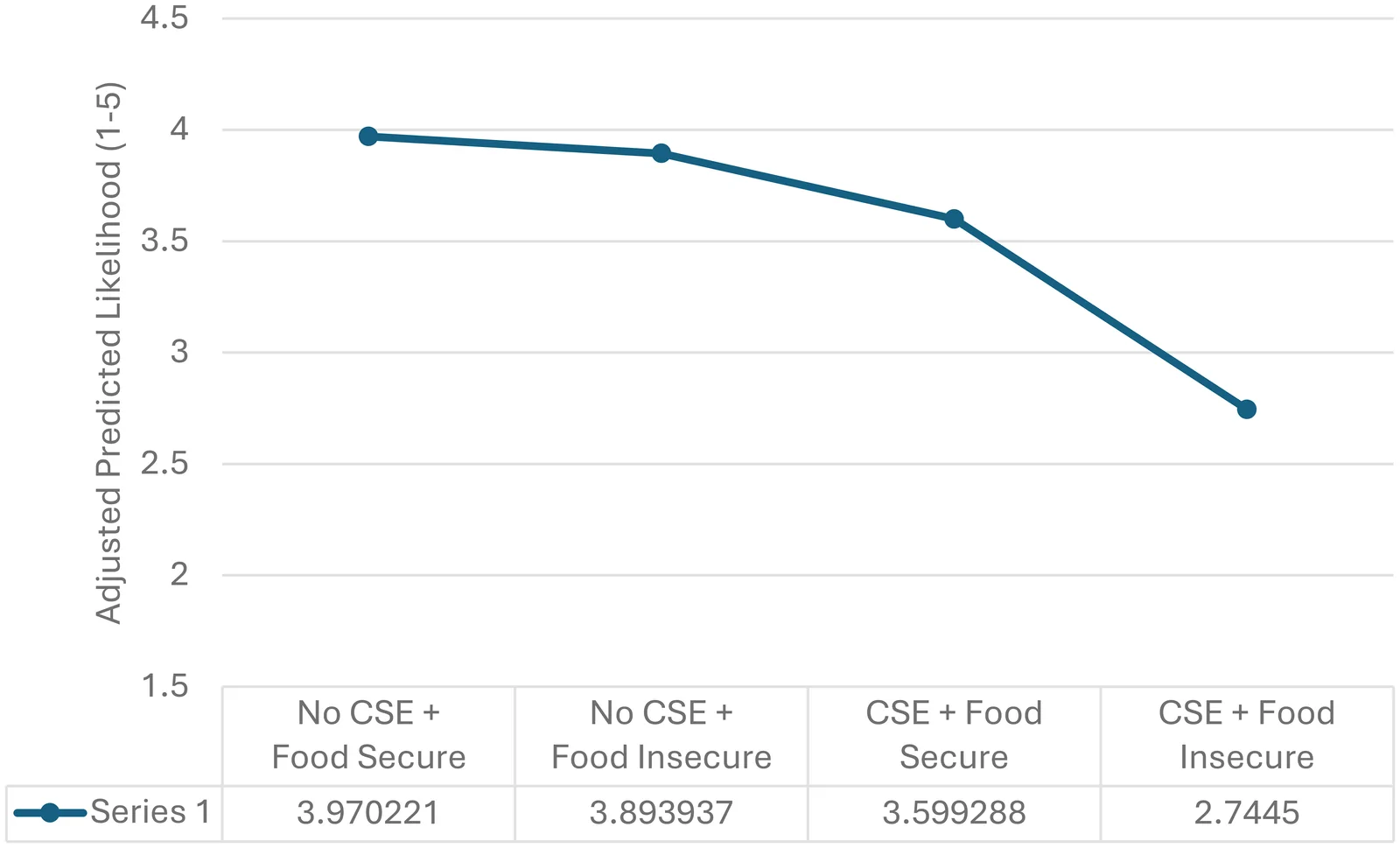
Parent disclosure likelihood across cumulative risk factors.

## Discussion

The findings presented here contribute to a growing body of research examining the relationship between structural vulnerability and commercial sexual exploitation (CSE) exposure (20, 43–46). The study found that food insecurity emerged as a particularly strong correlate of CSE exposure, and that both CSE exposure and food insecurity were related to reduced help-seeking self-efficacy and decreased intention to seek help if faced with a request to trade sex for money or something of value. The lower likelihood of help-seeking for these participants was driven primarily by a lower self-reported likelihood of seeking help from a parent or trusted adult. Findings highlight the enhanced vulnerability of youth who report food insecurity in combination with CSE exposure, and the need for youth in these conditions to be provided with education about extra-familial helping resources in the community.

### CSE exposure and food insecurity

Youth reporting CSE exposure were substantially more likely to experience food insecurity than youth without CSE exposure, despite no observed differences in broader measures of financial stress. This pattern may suggest that immediate unmet basic needs are more proximally associated with exploitation vulnerability than generalized perceptions of economic hardship. Findings are consistent with prior research identifying material instability and survival needs as important contextual vulnerabilities associated with exploitation risk among adolescents and young adults (22). More broadly, these findings align with ecological and resilience-oriented frameworks emphasizing the role of structural context in shaping youth vulnerability and access to protective resources, including help seeking (47).

The study did not find similar effects for the other measure of financial insecurity: family financial stress. This construct was assessed with only one question: whether they or their family did not have enough money to pay one or more bills in the prior month. It is possible that this single-item measurement lacked sensitivity to distinguish families that were experiencing significant financial hardship (e.g., youth may not have known the ability of their parents to pay bills). It is also possible that food insecurity is a strong marker for financial insecurity, and something youth in particular are aware of and affected by compared to other manifestations of financial insecurity. Prior research has found that food insecurity is related to, but not synonymous with poverty (25).

### Help-seeking and CSE risk

The study results also identified that CSE exposure is associated with lower confidence in help-seeking across a range of exploitative or threatening situations. Compared to non-exposed youth, CSE-exposed youth reported significantly lower confidence that they would know where to seek help if they felt unsafe at a party or were being pressured to trade sex. Similar patterns were observed in response to threats involving sexual images, although this difference did not reach statistical significance. Collectively, these findings suggest that youth with exploitation exposure may experience greater uncertainty navigating support systems during periods of elevated risk. Reduced help-seeking confidence may reflect prior negative experiences with disclosure, distrust of adults or institutions, normalization of exploitative experiences, or broader social isolation (48, 49). It may also indicate that the help-seeking confidence of CSE-exposed youth has been tested through experience, while for non-CSE-exposed youth, their help-seeking confidence is hypothetical. This could lead to inflated confidence. Importantly, even where findings were not statistically significant, associations were consistently in the direction of lower confidence and reduced help-seeking among CSE-exposed youth. These findings are also consistent with emerging research suggesting that youth impacted by exploitation may encounter barriers to formal disclosure and uncertainty regarding available support systems (40, 50).

Results from the current study suggest that differences in help-seeking likelihood for youth with CSE exposure appeared to be driven primarily by lower willingness to seek support from parents or other trusted adults. Specifically, while there were no significant differences between groups in the likelihood of seeking help from peers, the internet, community agencies, or emergency services, youth with CSE exposure reported substantially lower likelihood of seeking help from a parent or trusted adult if pressured to trade sex. This finding is notable given the well-established protective role that supportive adult relationships play in buffering against exploitation and other forms of victimization (51, 52). For some youth, parents or caregivers may not be perceived as emotionally safe, available, or trustworthy sources of support. Other youth may lack access to supportive adults altogether. In contexts characterized by family instability, unmet basic needs, or prior victimization, youth may become increasingly reliant on peers or individual coping strategies rather than adult-centered support systems (53, 54). These findings are consistent with broader literature suggesting that youth experiencing exploitation-related risks frequently rely on informal support networks and may be reluctant to engage formal systems or authority figures (40, 41).

Finally, results from cumulative risk analyses suggest that the accumulation of vulnerabilities such as food insecurity and CSE exposure may be important for understanding willingness by youth to seek support from trusted adults. Neither food insecurity alone nor CSE exposure alone was significantly associated with the likelihood of seeking help after adjustment for demographic characteristics. However, youth experiencing both food insecurity *and* CSE exposure reported significantly lower likelihood of seeking help from a parent or trusted adult, and analyses identified a significant linear trend across cumulative risk categories. The co-occurrence of CSE exposure and food insecurity could reflect a broader disconnection from stable support systems, increasing barriers to trusted supports and interventions.

### Limitations

Several limitations should be considered when interpreting these findings. First, the relatively small sample limited statistical power and increased the likelihood of Type II error. For example, multiple findings were consistent in direction but did not reach statistical significance. This suggests that some observed patterns may become clearer in terms of both clinical and statistical significance should the study be replicated in larger samples. Second, also related to the small sample size, we needed to examine only a composite CSE exposure measure and could not separate out indirect and direct exposure. Third, the cross-sectional design precludes conclusions regarding temporality or causality. It is not possible to determine whether food insecurity contributed to exploitation vulnerability, whether exploitation exacerbated instability and social disconnection, or whether these experiences developed reciprocally over time. Fourth, measures relied on self-report and may have been influenced by recall bias or social desirability concerns, particularly given the sensitive nature of exploitation-related experiences. Finally, the study assessed anticipated help-seeking intentions rather than observed behavior, and youths’ actual responses during exploitative situations may differ from hypothetical reporting patterns.

### Implications for research and practice

Findings from the study suggest that youth at greatest risk for exploitation may simultaneously be among the least likely to seek help from adults positioned to intervene. These findings have important implications for prevention and early intervention efforts. Youth-serving systems, including schools, healthcare settings, and community-based organizations, should recognize food insecurity and related indicators of structural vulnerability as potential markers of elevated exploitation risk of CSE. Prevention efforts focused exclusively on trafficking identification may overlook youth experiencing precursor conditions associated with cumulative exploitation vulnerability (55, 56). Nonetheless, early evaluation research has found that trafficking education programs can improve help-seeking likelihood in youth participants when compared to control groups (57). This information might be particularly relevant to youth in the vulnerable status highlighted by the current study. Future evaluation research should help ensure that trafficking prevention education results in improved help-seeking intention and behavior for youth with higher risk backgrounds.

Findings underscore the importance of strengthening youths’ access to safe and supportive adults outside of immediate family systems. For youth who do not perceive parents or caregivers as safe sources of support, other adults such as teachers, school counselors, mentors, coaches, healthcare providers, and community advocates may serve critical protective functions (47). Efforts to prevent exploitation should therefore include not only education regarding exploitation risk, but also intentional strategies to help youth identify trusted adults and strengthen supportive relationships within their broader social environments. Finally, increasing youth confidence in help-seeking behavior alone is not enough. When a young person seeks help, it is also essential that the help they receive is actually helpful. Future research should improve our understanding of how vulnerable youths’ experiences with help-seeking in the past shape subsequent help-seeking intention and behavior.

## Conclusions

The current study contributes to a limited but growing literature examining how structural vulnerability shapes help-seeking trajectories among youth exposed to exploitation-related risks. Findings highlight the importance of considering the availability of basic needs such as food alongside interpersonal and behavioral indicators of exploitation vulnerability. Further, this study points to the need to scaffold and support youth in help-seeking behaviors to increase knowledge and confidence in reaching out for appropriate help. Prevention and intervention efforts should prioritize not only identification of youth at elevated risk of CSE, but also the development of accessible and trustworthy support systems capable of engaging youth who may be reluctant to seek help through traditional familial pathways.

## Data Availability

The raw data supporting the conclusions of this article will be made available by the authors, without undue reservation.
